# Emotional Health and Mortality of Older Parents of Children With Serious Developmental Disabilities

**DOI:** 10.1093/geroni/igaa057.1478

**Published:** 2020-12-16

**Authors:** Jessica Hoyle, Sarah Laditka, James Laditka

**Affiliations:** 1 University of North Carolina - Charlotte, Charlotte, North Carolina, United States; 2 University of North Carolina at Charlotte, Charlotte, North Carolina, United States

## Abstract

Caring for a child with serious developmental disability (SDD) involves stress as parents age and anticipate the child’s future welfare. There are few studies of older parents of children with SDD. We followed parents for 18 years using the Panel Study of Income Dynamics (PSID). We defined SDD as: (1) autism, intellectual disability, learning disorder, epilepsy with seizures, attention deficit disorder/attention deficit hyperactivity disorder, or cerebral palsy; requiring (2) qualification for services and serious lasting impairment evidence. We used the PSID and its Child Development Supplement (1997-2007, 2014), linking children’s and parents’ data, with 5,780 parent-child dyads and 45,534 analytic observations. Parent outcomes included physician-diagnosed anxiety or depression, psychological distress (Kessler K6), and death. Discrete-time hazard analysis controlled for child and parent characteristics, and survey design. We identified parents of: Group 1, children without SDD or challenging behaviors; Group 2, SDD without challenging behaviors; Group 3, challenging behaviors without SDD; Group 4, SDD with challenging behaviors. Among parents ages 60+ and compared to Group 1, Group 2 through 4 odds ratios for anxiety/depression were, respectively, 1.28 (95% confidence interval, 0.63-1.92), 1.79 (1.35-2.24), and 2.62 (2.25-2.99), p-trend <.0001. Group 4 mental health risks were particularly high when children lived with the older parent: for anxiety/depression OR 6.75 (3.00-15.21), and distress OR 8.83 (3.58-21.77). At ages 60+ mortality was higher for parents of children with SDD (relative risk, RR 2.38, 1.21-4.67), especially Group 4 (RR 4.64, 1.39-15.47). Parents of children with developmental disabilities need emotional support, respite, and interventions addressing challenging behaviors.

